# THE ASSOCIATION BETWEEN VITAMIN K STATUS AND CARDIOVASCULAR DISEASE IN OLDER ADULTS

**DOI:** 10.1093/geroni/igz038.2393

**Published:** 2019-11-08

**Authors:** M Kyla Shea, Daniel Weiner, Gergory Matuszek, Sarah L Booth, Stephen Kritchevsky, Mary Cushman, Kathryn Barger

**Affiliations:** 1 Tufts University USDA Human Nutrition Research Center on Aging, Boston, Massachusetts, United States; 2 Tufts Medical Center, Boston, Massachusetts, United States; 3 Wake Forest School of Medicine, Winston-Salem, North Carolina, United States; 4 University of Vermont Larner College of Medicine, Burlington, Vermont, United States

## Abstract

A role for vitamin K in cardiovascular disease (CVD) has been proposed because vitamin K-dependent proteins are present in vascular tissue. We evaluated the association between vitamin K status and incident CVD and mortality in older adults from the Health, Aging, and Body Composition Study (Health ABC), and conducted a replication analysis using the Multi-ethnic Study of Atherosclerosis (MESA). In both cohorts circulating phylloquinone (vitamin K1), measured from baseline fasting blood samples, was categorized as ≤0.5nM, >0.5-≤1.0nM, and >1.0nM. Multivariable Cox proportional hazards models assessed the association between circulating phylloquinone and risk of a composite of CVD and mortality. In Health ABC (n=1246, mean age 74 years, 57% female, 58% Caucasian), over a median 11.5 follow-up years, participants with ≤0.5 nM plasma phylloquinone (n=351) had a 27% higher risk for CVD and mortality compared to those with >1.0nM (n=457) [adjusted hazard ratio (95% confidence interval) (HR(95%CI)): 1.27(1.06-1.52)]. However, the risk for CVD and mortality did not differ between those with >0.5-≤1.0nM (n=438) and with >1.0nM plasma phylloquinone [HR(95%CI): 1.03(0.87-1.52)]. Serum phylloquinone was similarly associated with CVD and mortality in MESA, over a median 12.1 follow-up years (n=764, mean age 62 years, 54% female, 35% Caucasian) [HR(95%CI), compared to those with >1.0nM (n=368): <0.5nM (n=253): 1.54(1.03-2.32); 0.5-≤1.0nM (n=153): 1.23(0.76, 1.98)]. Lower circulating phylloquinone was associated with a higher CVD and mortality risk in two independent cohorts. Additional studies are needed to corroborate our findings and clarify if certain segments of the population can derive cardiovascular benefit from improving vitamin K status.

